# Starting length and temperature dependence of eccentric muscle force–velocity behaviour

**DOI:** 10.1242/jeb.252267

**Published:** 2026-06-04

**Authors:** Roger W. P. Kissane, Graham N. Askew

**Affiliations:** ^1^ Department of Musculoskeletal and Ageing Science, University of Liverpool, The William Henry Duncan Building, 6 West Derby Street, Liverpool L7 8TX, UK; ^2^ School of Biomedical Sciences, University of Leeds, Leeds LS2 9JT, UK

**Keywords:** Force–velocity relationship, Eccentric contraction, Titin, Muscle mechanics, Musculoskeletal model

## Abstract

The force–velocity relationship underpins much of our understanding of muscle force generation during dynamic movements and is a component of musculoskeletal models. This relationship comprises a concentric (shortening) component and an eccentric (lengthening) component. While the concentric force–velocity relationship has been comprehensively described, and the importance of experimental conditions (e.g. temperature, starting length and fitting equations) aptly determined, there is no standardised approach to describe and quantify the complex relationship during eccentric contractions. Despite more than five decades of research, inconsistent starting lengths and temperature protocols limit the generalisation of findings across studies, constrain understanding of underlying mechanisms and hinder accurate integration of eccentric contractions into musculoskeletal models. Here, we have investigated the functional implications of different starting lengths and temperatures on the dynamic force–velocity relationship in the mouse soleus muscle. We show that the initial rapid rise in force (phase-1, cross-bridge dependent) is highly sensitive to the starting position on the force–length relationship, suggesting that the rate of force development is linked to the degree of actin–myosin overlap. The phase-1 response is also significantly affected by temperature, with lower temperatures reducing the rate of force development. Further, our data highlight that the second shallower force response (phase-2, non-contractile serial elastic component) is also significantly impacted by temperature, again reducing the rate of force development, probably through reduced/slower Ca^2+^ activation of titin. Together, these findings establish a framework for representing the dynamic behaviour of muscle during eccentric contractions, enabling the incorporation of physiologically realistic eccentric muscle properties into musculoskeletal models.

## Teaser

**Summary:** Eccentric muscle force development is highly sensitive to starting length and temperature, revealing distinct effects on contractile and elastic components and providing a framework for more realistic musculoskeletal modelling.

## INTRODUCTION

During deceleration, muscles absorb mechanical work (negative power), functioning as a brake (Daley and Biewener, 2011; Kissane et al., 2024). This energy may be dissipated directly by eccentric muscle fascicle lengthening (Kissane et al., 2024), or modulated by compliant tendons (muscle–tendon unit, MTU) that transiently absorb mechanical energy and then release it more gradually, stretching active muscle fascicles and reducing the rate at which energy is dissipated by the fibres (Konow and Roberts, 2015). The temporal profile of energy dissipation by actively lengthened muscle fascicles is constrained by their eccentric force–velocity relationship (Katz, 1939).

**Fig. 1.**
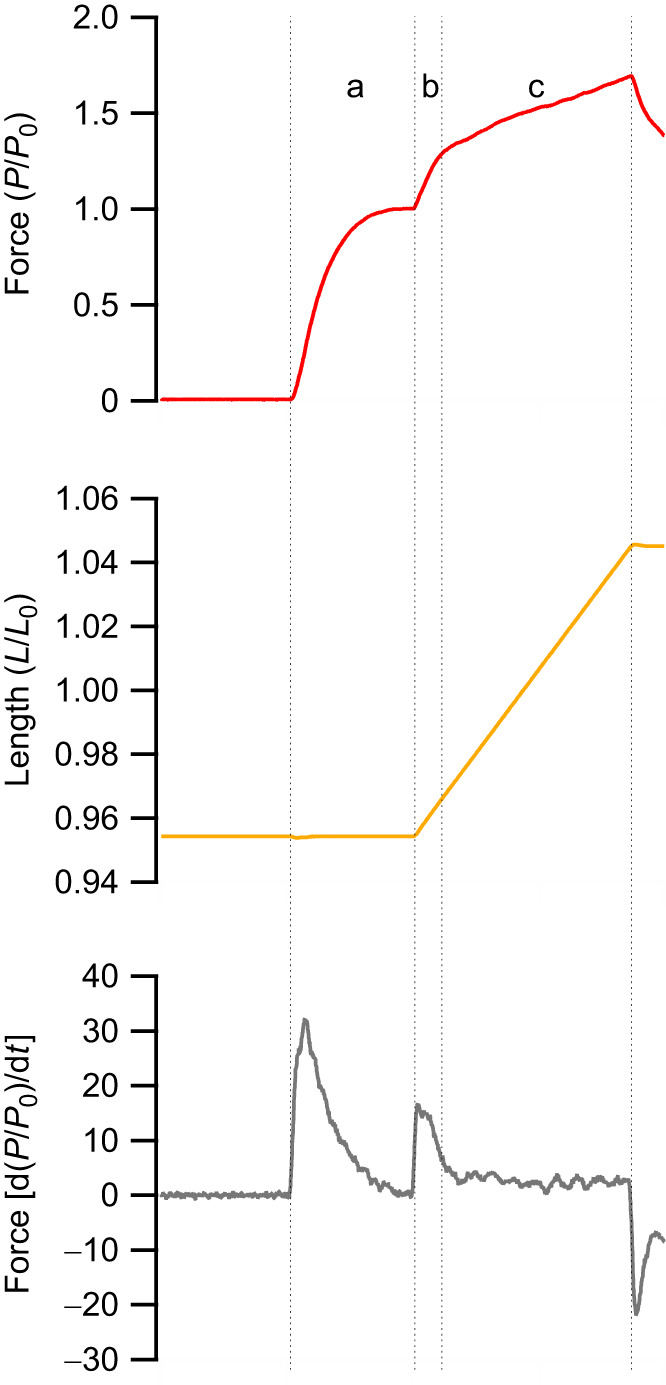
**Biphasic force response to lengthening.** Example muscle stimulated to generate a tetanus (a); the muscle stretch is imposed while holding the tetanic force. There is an immediate rapid increase in force (b), which we refer to as phase-1. This is followed by a drop in the rate of force development (c) and a constant, shallower maintenance in force development (phase-2).

Muscles undergoing active lengthening present with a dynamic force response with an initial rapid and steep phase-1 response followed by a longer and shallower phase-2 response (Josephson and Stokes, 1999; Joyce et al., 1969; Pinniger et al., 2006) (Fig. 1). The phase-1 response is thought to arise from elevated strain of attached cross-bridges (Flitney and Hirst, 1978; Linari et al., 2004). The abrupt detachment of myosin heads during phase-1 leads to the transition into the shallower phase-2 force response (Josephson and Stokes, 1999). The phase-2 force response is thought to be linked to increased strain of non-crossbridge, parallel elastic elements such as titin, which act as activation-dependent viscoelastic springs (Ramsey et al., 2010; Tahir et al., 2020; Tomalka, 2023).

An accurate understanding of the dynamic response of muscle to active stretch is essential for computational biomechanical models that aim to capture complex biological systems, predict *in vivo* muscle function and investigate optimal performance (Dick et al., 2017). The lack of standardisation in the methodologies to quantify this dynamic eccentric force–velocity relationship and uncertainty regarding the role of different experimental variables limits our ability to effectively describe this relationship (Askew and Kissane, 2026; Kissane, 2026). For example, different force profiles are obtained depending on whether measurements are made using isovelocity (velocity clamped; controlling the velocity of lengthening while observing the dynamic force response) or isotonic (force clamped; controlling the force levels and allowing the muscle to dynamically alter the velocity) contractions (Woledge et al., 1985). Other methodological variables include the starting length of the muscle relative to the force–length relationship, and the total strain applied, which are typically not standardised (Tahir et al., 2020). Some studies use a range of strains and different starting positions on the force–length relationship, which may be important because the overlap between thick and thin filaments can critically affect the rate of force development (Krylow and Sandercock, 1997).

The effects of temperature on the concentric force–velocity relationship have been characterised extensively (Bennett, 1984, 1985; Ranatunga, 2018; Rummel et al., 2021); however, the sensitivity of the eccentric force–velocity relationship to temperature is less well known. Given that the phase-1 force response to muscle lengthening is underpinned by cross-bridge detachment kinetics, it is highly likely that this component of the dynamic force response to active stretch is temperature sensitive (Askew and Kissane, 2026; Colombini et al., 2008; Roots et al., 2012). Further, the phase-2 response is determined by parallel elastic mechanisms, such as titin–actin binding (Hessel et al., 2017; Monroy et al., 2017; Tahir et al., 2020). Given that the eccentric force–velocity relationship has been characterised in many mammalian muscles at non-physiological temperatures (5–25°C; Minozzo and Rassier, 2010; Ramsey et al., 2010; Tomalka et al., 2017; Weidner et al., 2022), understanding its sensitivity to temperature is crucial.

The lack of standardisation of experimental protocols for investigating the force response to active stretch together with uncertainty over the importance of key methodological parameters makes it difficult to assess the validity of current modelling approaches and refine them in a substantiated manner. The default eccentric force–velocity characterisation implemented by Millard et al. (2013), widely used in musculoskeletal models, is likely to be sub-optimal. Their modelling approach was based on data from an *in situ* preparation of the cat soleus muscle (Joyce et al., 1969) at 37°C and *ex vivo* measurements from the semitendinosus muscle of the frog (Mashima et al., 1972) in which the muscles were subjected to different strains, and operated at different positions on the force–length relationship. Consequently, averaging these datasets may be inappropriate and underestimate the plateau of the eccentric force–velocity relationship in mammalian muscle (Charles et al., 2024; Kissane and Askew, 2024). Newer, more complex models have been developed including the extended Hill-type model which includes the viscoelastic tendon and the viscoelastic crossbridge and active titin elements (Millard et al., 2024). These models still vary in their ability to accurately predict the dynamic biphasic shape of the eccentric response.

The aim of the study was to investigate how ramp starting length and temperature influence the dynamic force response of a mammalian skeletal muscle during maximal active lengthening contractions. Using the mouse soleus muscle (SOL) in an *ex vivo* set up (Charles et al., 2024; Kissane and Askew, 2024), we varied both ramp starting length and temperature (17, 27 and 37°C). Eccentric ramps were initiated from different starting positions on the ascending limb of the force–length relationship and crossed the plateau. We hypothesised that: (1) the rate of force development during phase-1 will be reduced at higher temperatures because of faster cross-bridge detachment; (2) the rate of force development during phase-1 will be reduced at shorter ramp starting lengths because of the reduced number of attached cross-bridges on the ascending limb (Krylow and Sandercock, 1997); and (3) the phase-2 response will show less sensitivity to temperature and ramp starting length as a consequence of it being predominantly determined by parallel elasticity rather than cross-bridge kinetics.

## MATERIALS AND METHODS

All experimental procedures complied with institutional and UK Home Office guidance. This study adheres to the ethical standards set by the journal and follows established guidelines for animal research (Percie du Sert et al., 2020).

### Animals

Twenty-seven male C57B6 mice (Charles River) weighing 25.4±2.0 g (approximately 12 weeks old) were used in this study. Animals were housed under a 12 h:12 h light:dark cycle at 21°C and had *ad libitum* access to food and water.

### *Ex vivo* soleus preparation

Experiments were conducted on the intact mouse soleus muscle, a predominantly fusiform, unipennate muscle–tendon unit. As such, the measured force responses primarily reflect behaviour at the whole-muscle level. We note that muscles with different architectures (e.g. bipennate or multipennate muscles) may exhibit different mechanical responses during active lengthening due to factors such as fibre rotation, variable pennation angles and heterogeneous strain distributions. Mice were euthanised using approved Schedule 1 methods, as defined in the UK Animals (Scientific Procedures) Act 1986. Both hindlimbs of the mouse were transferred to chilled (4°C), oxygenated (95% O_2_, 5% CO_2_) Krebs–Henseleit solution (mmol l^−1^; 117 NaCl, 4.7 KCl, 2.5 CaCl_2_, 1.2 MgSO_4_, 24.8 NaHCO_3_, 1.2 KH_2_PO_4_ and 11.1 glucose) (Burton, 1975). The SOL was dissected free from both hindlimbs and aluminium clips were attached to the proximal and distal ends (Askew and Marsh, 1997). The experimental muscle was suspended vertically in a flow-through Perspex chamber filled with circulating, oxygenated Krebs–Henseleit solution, while the second SOL was left in oxygenated Krebs–Henseleit solution at 4°C until required, pinned out at approximately resting length. Muscles were attached to an ergometer (series 300B-LR, Aurora Scientific Inc.; 1 N force range, 10 mm excursion) via a stainless-steel rod and muscle length was altered using a digital height gauge (Mitutoyo Corporation, Kanagawa, Japan). Muscles were left for 30 min to thermo-equilibrate and recover from the dissection. Platinum electrodes were placed inside the chamber on either side and parallel to the long-axis of the muscle and the muscle was activated using an isolated stimulator (Universal Isolated Stimulator Output, Hugo Sachs Elektronik, Harvard Apparatus GmbH, March-Hugstetten, Germany).

### Isometric characteristics

All muscles were subjected to a series of isometric twitches (supramaximal stimulus of 0.2 ms pulse) and muscle lengths were incrementally increased to find the optimal length of force generation (*L*_0_). Maximal isometric tetanic force at *L*_0_ was determined using a train of stimuli delivered at an optimised frequency of stimulation (to generate a fully fused tetanus) for each muscle, at each temperature. Data were sampled at 2 kHz during isometric twitch kinetics. Periodically, control isometric tetanic contractions were performed to monitor the condition of the muscle. Data were excluded (and the experiment terminated) if the control maximum isometric tetanic force (*P*_0_) fell below 70% of the maximum value recorded (Kissane et al., 2022, 2018; Warren et al., 2020).

### Experiment 1 – effects of ramp starting length on eccentric dynamic force response

In these experiments, the SOL were maintained at 37°C using a continuous flow of oxygenated Krebs–Henseleit solution, maintained by a water bath [LT ecocool 100, Grant Instruments (Cambridge) Ltd, Royston, Herts, UK]. Isovelocity (approximately 3%, 6%, 12%, 18%, 25% and 50% *V*/*V*_max_) eccentric ramps were initiated at different relative starting lengths on the ascending limb of the force–length relationship (0.95, 0.9 and 0.85 of *L*_0_) and extended beyond the plateau (overall strain 10%, 20% or 30% of *L*_0_, respectively) (Kissane and Askew, 2024).

### Eccentric force–velocity characteristics

The eccentric force–velocity relationship was determined using isovelocity lengthening ramps (Fig. 1). The SOL were lengthened by 2 mm, symmetrically spanning *L*_0_ (Kissane and Askew, 2024) representative of mean±s.d. starting lengths of 0.906±0.006 *L*/*L*_0_ (17°C), 0.902±0.005 *L*/*L*_0_ (27°C) and 0.900±0.006 *L*/*L*_0_ (37°C), which are not significantly different from one another (ANOVA *F*_2,18_=1.800, P=0.194). Unlike during isotonic concentric contractions, in which force and velocity have a single corresponding value, the force response during isovelocity eccentric contractions is dynamic. To standardise the approach and follow convention, force and velocity were determined over a length change spanning *L*_0_, corresponding to the phase-2 response, and averaged over a 0.05 mm window centred on *L*_0_. Muscles were lengthened at velocities up ∼60% of *V*_max_, with data sampled at 5 kHz. We also measured the relative force (*P*/*P*_0_) at the transition between the linear phase-1 slope and phase-2 (analogous to the critical point in Minozzo and Rassier, 2010). The lengthening force–velocity relationship was then fitted using a hyperbolic function (Alcazar et al., 2019; Kissane and Askew, 2024) (Eqn 1). Coefficients *D* and *E* were derived which describe the plateau height (*D*) and curvature (*E*) of the relationship (Kissane and Askew, 2024) (Fig. S1):
(1)

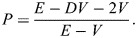


### Experiment 2 – temperature effects on contractile properties

Muscles were maintained at 17, 27 or 37°C throughout the course of the experiment using a continuous flow of oxygenated Krebs–Henseleit solution, maintained by a water bath [LT ecocool 100, Grant Instruments (Cambridge) Ltd].

### Concentric force–velocity characteristics

The concentric force–velocity relationship of the SOL was determined using a series of isotonic afterload contractions across a range of forces (5–80% of *P*_0_; Kissane et al., 2018), with data sampled at 2 kHz. The concentric force–velocity relationship was determined by fitting a hyperbolic-linear function (Marsh and Bennett, 1986) to the data (Eqn 2):
(2)

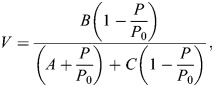
where *P*/*P*_0_ is relative force and *A*, *B* and *C* are coefficients.

The maximal shortening velocity at zero force (*V*_max_), expressed relative to fibre length, was calculated, assuming a relative fibre length of 0.85 *L*_0_ (Askew and Marsh, 1997). Peak instantaneous isotonic power 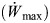 and the power ratio (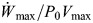; a measure of the curvature of the concentric force–velocity relationship) were also determined from the fitted concentric force–velocity relationship.

### Eccentric force–velocity characteristics

As above for experiment 1, force and velocity were determined over a length change spanning *L*_0_, corresponding to the phase-2 response, and were averaged over a 0.05 mm window centred on *L*_0_ and at the transition point between phase-1 and phase-2. Muscles were actively lengthened at velocities up ∼60% of *V*_max_, and data sampled at 5 kHz.

Muscle ‘give’, is a fibre kinetic phenomenon which occurs during active muscle lengthening. It is characterised by a sharp drop in force immediately after the phase-1 component, followed by the phase-2 shallower, steady lengthening response (Tomalka, 2023). Eccentric ramps were binarily categorised into groups that presented with or without muscle ‘give’, determined by the presence of a transient drop in force, below that of the transition point.

### Statistics

One-way ANOVA were used to assess statistical significance in isotonic and isovelocity derived force–velocity metrics and coefficients in the fitted relationships. Where significance was detected, *post hoc* comparisons were made using the Bonferroni correction and the threshold for statistical significance was set to *P*<0.05 (completed using SPSS 28, v28.0.1.1). Linear data comparisons were computed in R (v4.5.0; https://www.r-project.org/). Repeated measures correlations (Bakdash and Marusich, 2017) were used to determine the overall within-individual relationship between rate of relative force development and lengthening velocity (relative to fibre length or *V*_max_). To test whether the relationship between velocity and force differed with eccentric ramp starting length (5%, 10% and 15% below *L*_0_), or temperature (17°C, 27°C and 37°C), a linear regression model was fitted with force (dependent variable), velocity (continuous predictor) and strain/temperature (categorical predictor). An interaction term between velocity and starting length/temperature was included to evaluate whether the slope of force on velocity varied with starting length/temperature. Estimated marginal trends (slopes) of velocity within each group were extracted using the emtrends function from the emmeans package (Searle et al., 1980). Pairwise comparisons of these slopes were performed using pairs, with Tukey-adjusted *P*-values to control for multiple comparisons. All data processing and regression and box plot figure generation was carried out using Igor Pro 8 (v8.0.4.2). Surface plots were created in Matlab (R2021a) using the surf function.

## RESULTS

### The effects of eccentric ramp starting length on the active lengthening biphasic force response

**Fig. 2.**
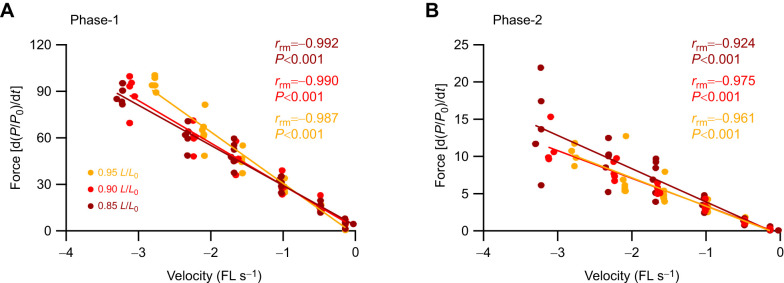
**Sensitivity of the eccentric phase-1 and phase-2 response to strain.** The relationship between lengthening velocity and the rate of relative force development for phase-1 (A) and phase-2 (B) in response to altered starting lengths: 0.95 *L*/*L*_0_ (*n*=6), 0.90 *L*/*L*_0_ (*n*=5), 0.85 *L*/*L*_0_ (*n*=5). FL, fibre length.

At all three ramp starting lengths there was a significant relationship between the rate of relative force development during phase-1 and absolute lengthening velocity (Fig. 2A: 0.95 *L*_0_, *r*_rm29_=−0.987, 95% CI [−0.9938, −0.9731], *P*<0.001; 0.90 *L*_0_, *r*_rm24_=−0.990, 95% CI [−0.9954, −0.9765], *P*<0.001; and 0.85 *L*_0_, *r*_rm25_=−0.992, 95% CI [−0.964, −0.9823], *P*<0.001). At each starting length there was a significant linear relationship between the rate of relative force development during phase-1 and lengthening velocity; the slope of this relationship varied with starting length (Fig. 2A; Table S1). At 0.95 *L*_0_, the rate of relative force development was significantly higher than at 0.90 *L*_0_ (*P*=0.0012) and 0.85 *L*_0_ (*P*<0.001). Similar to phase-1, at all three ramp starting lengths, there was a significant relationship between the rate of relative force development during phase-2 and the absolute lengthening velocity (Fig. 2B: 0.95 *L*_0_, *r*_rm29_=−0.961, 95% CI [−0.9811, −0.9196], *P*<0.001; 0.90 *L*_0_, *r*_rm24_=−0.975, 95% CI [−0.9888, −0.9437], *P*<0.001; and 0.85 *L*_0_, *r*_rm25_=−0.924, 95% CI [−0.9651, −0.8384], *P*<0.001). The slope of the relationship between the rate of relative force development during phase-2 and lengthening velocity was not significantly different across the three starting lengths (Table S1).

### The effects of eccentric ramp starting length on the eccentric force–velocity relationship

**Fig. 3.**
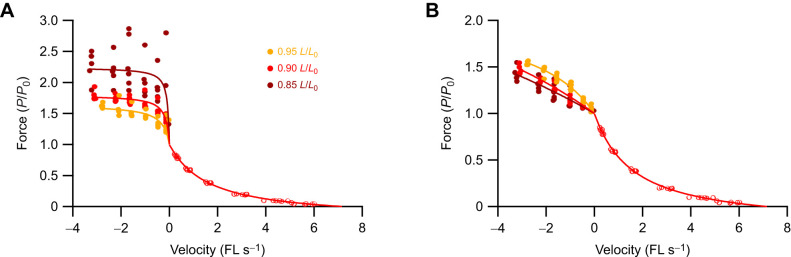
**Sensitivity of the eccentric force–velocity relationship to strain.** The force–velocity relationship derived from values at *L*_0_ (A) and at the transition point between phase-1 and phase-2 (B) at different starting lengths: 0.95 *L*/*L*_0_ (*n*=6), 0.90 *L*/*L*_0_ (*n*=5), 0.85 *L*/*L*_0_ (*n*=5).

The coefficients of the equation fitted to the eccentric force–velocity relationship (Kissane and Askew, 2024) correspond to different dynamic features of the eccentric force–velocity curve: coefficient *D* corresponds to plateau height and coefficient *E* to the curvature of the relationship (Kissane and Askew, 2024; Fig. S1). The *D* coefficient measured at *L*_0_ was significantly affected by ramp starting length (ANOVA *F*_2,12_=6.350, *P*<0.001; Table 1, Fig. 3A), with lower plateau heights for ramps starting at 0.95 *L*_0_ compared with 0.85 *L*_0_ (*P*<0.001). Similarly, the *E* coefficient (ANOVA *F*_2,12_=6.627, *P*=0.012; Table 1) was significantly different across the three starting lengths, with the curvature for ramps starting at lengths of 0.95 *L*_0_ being significantly lower than that at starting lengths of 0.90 *L*_0_ (*P*=0.016). Interestingly, despite the starting position of the muscle lengthening on the force–length relationship (Fig. S2), both these coefficients were not significantly different when taken from the transition point between phase-1 and phase-2 (Table 1, Fig. 3B).

**Table 1.** Eccentric force–velocity curve coefficients in relation to strain in the soleus muscle

	0.95 *L*/*L*_0_	0.90 *L*/*L*_0_	0.85 *L*/*L*_0_	*F*	*P*
At *L*_0_					
* D*	−0.316±0.040^‡^	−0.204±0.085	0.187±0.241*	17.937	<0.001
* E*	0.418±0.140^§^	0.152±0.033*	0.195±0.182	6.627	0.012
At transition point					
* D*	0.230±0.145	4.193±5.472	1.629±3.623	1.837	0.198
* E*	3.396±0.719	30.199±33.339	18.454±23.487	1.941	0.183

*D* and *E* correspond to the value fitted for the hyperbolic equation of Kissane and Askew (2024) to fit the eccentric portion of the force–velocity relationship, displayed in Eqn 2. Data are means±s.d.: *P<0.05 vs 10% (*n*=6), ^§^*P*<0.05 vs 20% (*n*=5), ^‡^*P*<0.05 vs 30% (*n*=5).

### The effects of temperature on the active lengthening biphasic dynamic force response

**Fig. 4.**
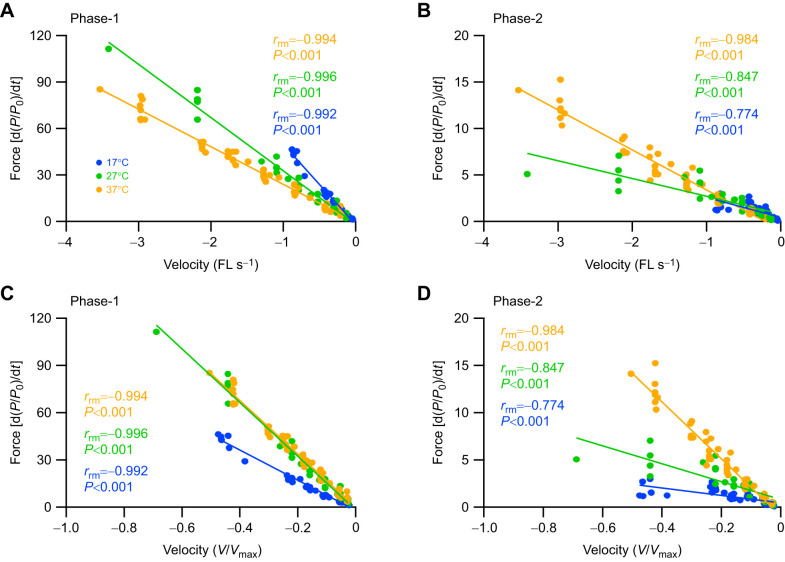
**Sensitivity of the eccentric phase-1 and phase-2 response to temperature.** The relationship between lengthening velocity and the rate of force development during phase-1 (A,C) and phase-2 (B,D), for absolute lengthening velocity (A,B) and velocity normalised to temperature-specific *V*_max_ (C,D) at different temperatures: 17°C (*n*=6), 27°C (*n*=5), 37°C (*n*=8).

At all three temperatures (17, 27 and 37°C) there was a significant relationship between the rate of relative force development during phase-1 and both absolute lengthening velocity (Fig. 4A) and velocity normalised to temperature-specific maximum shortening velocity (Fig. 4C) (17°C, *r*_rm28_=−0.992, 95% CI [−0.9961, −0.9825], *P*<0.001; 27°C, *r*_rm24_=−0.996, 95% CI [−0.9982, −0.9908], *P*<0.001; and 37°C, *r*_rm44_=−0.994, 95% CI [−0.9968, −0.9896], *P*<0.001). The slope of these relationships for phase-1 was significantly affected by temperature (Table S2). Relative to absolute lengthening velocity, the slope at 17°C was significantly higher than at 27°C (Fig. 4A; *P*<0.001) and at 37°C (Fig. 4A; *P*<0.001); however, relative to velocity normalised to temperature-specific *V*_max_, this relationship was reversed (Fig. 3B; Table S2).

Similar to phase-1, there was a significant relationship between the rate of relative force development during phase-2 and both the absolute (Fig. 4B) and normalised (Fig. 4D) lengthening velocity at all three temperatures (17°C, *r*_rm28_=−0.774, 95% CI [−0.8867, −0.5730], *P*<0.001; 27°C, *r*_rm24_=−0.847, 95% CI [−0.9292, −0.6834], *P*<0.001; and 37°C, *r*_rm44_=−0.984, 95% CI [−0.9909, −0.9703], *P*<0.001). The slopes of these relationships were significantly affected by temperature (Table S2). At 17°C, the slope was significantly shallower than at 37°C when plotted against absolute lengthening velocity (Table S2; *P*<0.001), and when normalised to temperature-specific *V*_max_ (Table S2; *P*<0.001).

### The effects of temperature on the concentric force–velocity relationship

**Fig. 5.**
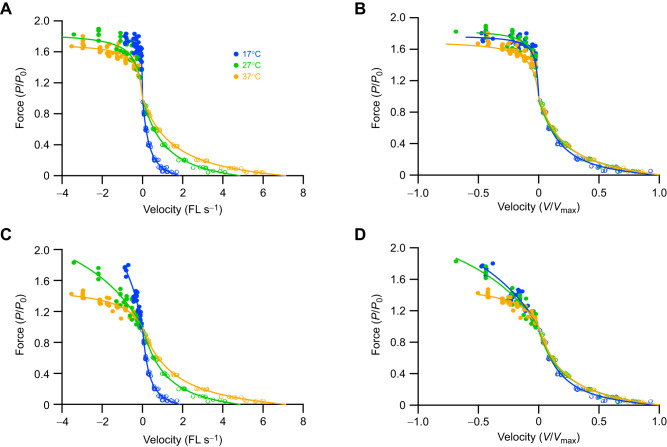
**Sensitivity of the force–velocity relationship to temperature.** The force–velocity relationship at 17°C (*n*=6), 27°C (*n*=5) and 37°C (*n*=8) for the soleus when measured as the muscle lengthens across *L*_0_ (A,B) and at the transition point between phase-1 and phase-2 (C,D), for absolute lengthening velocity (A,C) and velocity normalised to temperature-specific *V*_max_ (B,D).

The temperature sensitivity of the concentric force–velocity relationship has been well characterised (Bennett, 1984; Ranatunga, 1984, 2018). However, to provide a basis for normalising the eccentric shortening velocities, we quantified this relationship for the SOL at each temperature (Table 2). As expected, the concentric force–velocity relationship was temperature dependent. The maximum shortening velocity (ANOVA *F*_2,18_=165.101, *P*<0.001; Table 2, Fig. 5A,C), and maximum isotonic power (ANOVA *F*_2,18_=31.725, *P*<0.001; Table 2) decreased significantly with decreasing temperature. The *D* coefficient presented with significant variability across temperatures (varying plateau height, ANOVA *F*_2,16_=6.350, *P*=0.009; Table 3, Fig. 5A,B), with 27°C presenting with the lowest *D* coefficient. The *E* coefficient significantly increased (reducing curvature, ANOVA *F*_2,16_=15.602, *P*<0.001; Table 3, Fig. 5C,D) when measured at *L*_0_ across the three temperatures. However, neither coefficient was significantly different when taken from the transition point between phase-1 and phase-2 (Table 3).

**Table 2.** Isotonic properties and coefficients for the concentric force–velocity relationship fit of the soleus muscle in relation to temperature

	17°C	27°C	37°C	*F*	*P*
*V*_max_ (FL s^−1^)	1.85±0.5^§,‡^	4.96±0.55*^,‡^	7.01±0.6*^,§^	165.101	<0.001
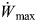 (W kg^−1^)	27.94±10.52^§,‡^	120.75±23.00*	119.97±32.60*	31.725	<0.001
*P*/*P_0_* at maximal power	0.30±0.03	0.33±0.02	0.30±0.02	1.833	0.189
*V*/*V*_max_ at maximum power	0.26±0.06	0.28±0.01	0.30±0.04	1.069	0.364
Power ratio	0.08±0.02	0.09±0.01	0.09±0.01	1.007	0.385
*A*	0.217±0.210	0.180±0.026	0.244±0.130	0.293	0.750
*B*	0.376±0.364^‡^	0.815±0.118	1.811±1.177*	6.503	0.007
*C*	0.023±0.257	0.406±0.217	−0.157±1.045	0.987	0.392

*V*_max_, maximum shortening velocity expressed relative to mean fibre length, FL; 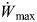, maximum isotonic power. Coefficients *A*, *B* and *C* correspond to values for the Marsh and Bennett (1986) hyperbolic linear fit for the concentric portion of the force–velocity relationship presented in Eqn 1. Data are means±s.d.: *P<0.05 vs 17°C (*n*=7), ^§^*P*<0.05 vs 27°C (*n*=5), ^‡^*P*<0.05 vs 37°C (*n*=9).

**Table 3.** Eccentric force–velocity curve coefficients in relation to temperature in the soleus muscle

	17°C	27°C	37°C	*F*	*P*
At *L*_0_					
D	−0.216±0.084	−0.123±0.046^‡^	−0.238±0.038^§^	6.350	0.009
E	0.035±0.016^§,‡^	0.243±0.142*	0.363±0.124*	15.602	<0.001
At transition point					
D	1.285±0.636	0.620±2.970	−0.430±0.106	2.236	0.139
E	1.729±0.731	3.696±10.923	1.309±0.502	0.308	0.739

*D* and *E* correspond to the value fitted for the hyperbolic equation of Kissane and Askew (2024) to fit the eccentric portion of the force–velocity relationship, displayed in Eqn 2. Data are means±s.d.: *P<0.05 vs 17°C (*n*=6), ^§^*P*<0.05 vs 27°C (*n*=5), ^‡^*P*<0.05 vs 37°C (*n*=8).

### The effect of temperature on muscle ‘give’

**Fig. 6.**
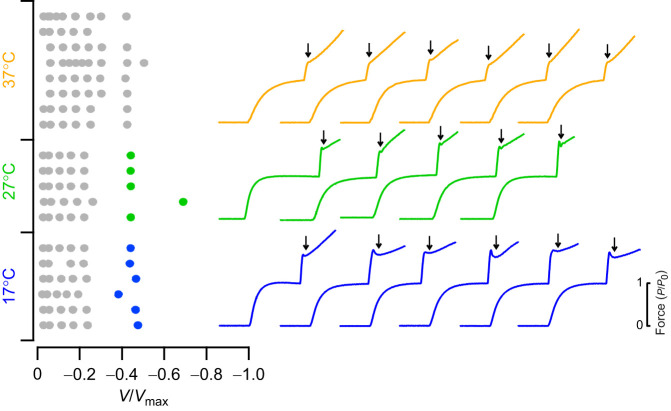
**The presence of ‘give’ during eccentric ramps.** Scatter plot of the *V*/*V*_max_ ramp applied to individual muscles (rows of circles). Coloured circles indicate the presence of muscle ‘give’; grey circles indicate no muscle ‘give’. Insets are raw traces for each of the fastest eccentric ramps for each temperature condition. Black arrows at the transition point highlight the lack of evidence for muscle ‘give’ at 37°C, with increasing depth of muscle ‘give’ as temperature decreases: 17°C (*n*=6), 27°C (*n*=5), 37°C (*n*=8).

A total of 118 eccentric ramps were examined across the temperature range (17°C, *n*=35; 27°C, *n*=30; and 37°C, *n*=53) to record the presence of muscle ‘give’ (Fig. 6). The two lower temperatures showed the highest frequency of muscle ‘give’ (17°C: 6/35; and 27°C: 5/30), whereas no occurrences were observed at 37°C (0/53).

## DISCUSSION

The eccentric force–velocity relationship remains challenging to quantify and describe accurately (Kissane and Askew, 2024), largely because of the dynamic nature of the force response during active stretch (Fig. 1). Over the past half-century, the mechanisms that underpin the dynamic force response to active stretch have begun to be revealed. However, these mechanisms are likely to be sensitive to variables such as temperature and ramp relative starting position on the force–length relationship. These experimental variables have not typically been standardised when quantifying the eccentric force–velocity relationship, and vary considerably across published studies. Consequently, it remains difficult to reconcile findings across the literature, limiting our ability to interpret the existing body of data and apply it robustly in musculoskeletal models.

**Fig. 7.**
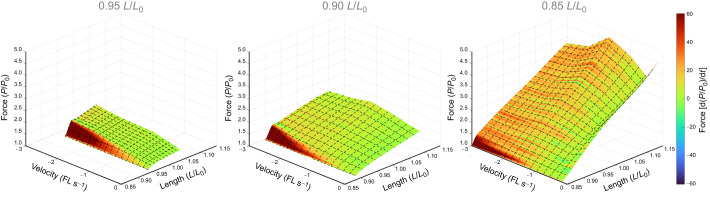
**The force–length–velocity surface plot at varying strains on the soleus.** The relationship between muscle lengthening velocity, and force production and length in response to altered starting length (*L*/*L*_0_). The heatmap is the rate of force development.

**Fig. 8.**
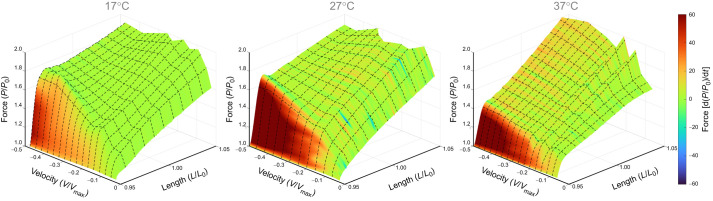
**The force–length**–**velocity surface plot at varying temperatures on the soleus.** The relationship between muscle lengthening velocity, and force production and length at 17, 27 and 37°C. The heatmap corresponds to the rate of force development.

In this study, we provide a comprehensive assessment of the sensitivity of the eccentric force–velocity behaviour to variation in starting length and temperature of the intact whole-mouse SOL. Specifically, we identify a strong dependence of the rate of relative force development during phase-1 on the initial ramp starting position on the force–length relationship. In addition, both phase-1 (cross-bridge-dependent component) and phase-2 (parallel elastic component) dynamic force responses are highly temperature sensitive. Building upon the force–length–velocity plots introduced by Josephson and Stokes (1999), we present the dynamic impact of eccentric ramp starting length (Fig. 7) and temperature (Fig. 8) on the interaction between force, length and velocity – an approach that may inform the development of more realistic musculoskeletal models (Yeo et al., 2023).

### The importance of ramp starting length on the dynamic force response to active stretch

There has been a significant lack of standardisation of the relative position on the force–length relationship at which the muscle is isometrically tetanised and the stretch initiated. While some studies have conducted ramps on the ascending limb of the force–length relationship (Krylow and Sandercock, 1997; Tahir et al., 2020), others have started the stretch at *L*_0_ (Flitney and Hirst, 1978; Ramsey et al., 2010). Despite a limited commentary on the length dependence of the rate of force development (Krylow and Sandercock, 1997), the implications of different levels of overlap between the thick and thin filaments on the subsequent eccentric force–velocity relationship have not been systematically investigated.

In this study, we characterised the dynamic relationship between force, ramp starting length and stretch velocity (Fig. 7). The sharp rise in force during phase-1 is thought to reflect strain of an attached cross-bridge population and exhibits an approximately linear dependence on lengthening velocity (Kissane and Askew, 2024; Tomalka, 2023). As hypothesised, initiating ramps at relatively shorter lengths on the ascending limb, where filament overlap and cross-bridge attachment probability are reduced (including potential length-dependent effects on lattice spacing; Hessel et al., 2024; Rassier and Månsson, 2024), lowered the rate of relative force development. Notably, the relationship between phase-1 rate of relative force development and lengthening velocity remained strongly linear across starting lengths, consistent with a common underlying strain-dependent mechanism whose gain is set by the available attached cross-bridge population and the mechanical conditions at stretch onset.

Following phase-1, the dynamic force response transitions to phase-2 as the highly strained cross-bridge population is rapidly lost (detached), producing a defined transition point (often termed the critical force in single-fibre studies). This transition shows strong velocity dependence (Kissane and Askew, 2024) (Figs 3B and 5B,D) and is likely to be influenced by the number of attached cross-bridges (Flitney and Hirst, 1978; Minozzo and Rassier, 2010; Pinniger et al., 2006). In our whole-muscle preparation, the relationship between the transition point and phase-1 stiffness (Fig. S3A) shifted with starting length, such that initiating stretch at shorter relative lengths on the ascending limb led to the transition occurring at lower absolute stiffness, potentially underpinned by starting length-dependent changes in mechanical heterogeneity, non-uniform strain and force distribution across fibres and serially connected sarcomeres/half-sarcomeres during the ramp (Rassier and Månsson, 2024).

Finally, the phase-2 response, where force generation is attributed to non-cross-bridge parallel elastic mechanisms (Ramsey et al., 2010; Tahir et al., 2020; Tomalka, 2023) was also affected by starting length. In this study, we systematically measured the rate of relative force development at *L*_0_; therefore, it is unsurprising that this rate did not differ significantly between the three ramp starting lengths investigated. Notably, the muscle was able to maintain force during the phase-2 response, even when subjected to a 30% strain starting at 0.85 *L*_0_, generating forces up to 4.5 times the maximum isometric tetanic force (Fig. 7).

### The importance of temperature on the dynamic force response to active stretch

There is considerable variation in the temperature at which mammalian muscle contractile characteristics are experimentally determined. In *in situ* experiments, muscles are typically maintained at (or close to) core body temperature (∼37°C; Kissane and Askew, 2024; Siebert et al., 2015). Conversely, isolated *ex vivo* preparations are often maintained at room temperature (Ramsey et al., 2010; Tahir et al., 2020) or core body temperature (Askew and Kissane, 2026), while skinned fibre experiments are conducted at even lower temperatures (∼5–11°C) (Linari et al., 2004; Minozzo and Rassier, 2010; Tomalka et al., 2017). The rationale for using non-physiological temperatures is generally to improve preparation longevity, and it is thought that isometric force may be similar across temperatures (Tahir et al., 2020; Weidner et al., 2022). However, this overlooks extensive evidence that skeletal muscle cross-bridge cycling is highly temperature dependent (Bennett, 1984, 1985; Ranatunga, 2018; Rummel et al., 2021), with lower temperatures specifically shown to reduce the relaxation state of the myosin motors and reduce the fraction of those that activate (Caremani et al., 2019; Caremani et al., 2021). Consequently, contractile properties during eccentric contractions are expected to be profoundly affected by temperature given their dependence on cross-bridge detachment kinetics, making it unreliable to relate characteristics measured at non-physiological temperatures directly to *in vivo* muscle behaviour.

Our findings reveal that temperature has a strong influence on the relationship between the rate of relative force development and stretch velocity during phase-1. At lower temperatures, the slope of this relationship is steeper, indicating greater stiffness of the muscle, as indicated by *Q*_10_ values below 1: 0.66 for 17°C to 27°C and 0.71 for 27°C to 37°C. This supports our hypothesis that elevated temperatures accelerate cross-bridge detachment during active stretch, thereby reducing stiffness. It is noteworthy that when stretch velocity is expressed relative to the temperature-specific maximum shortening velocity, the trend reverses and the slope becomes shallower at lower temperatures, suggesting reduced stiffness, indicated by *Q*_10_ values above 1: 1.77 for the 17°C to 27°C range. As noted previously (Askew and Kissane, 2026), this pattern implies that at low temperatures, mechanical strain-dependent detachment mechanisms play an important role alongside cross-bridge detachment kinetics.

Interestingly, despite the lower rate of relative force development during phase-1, the transition to phase-2 is delayed at lower temperatures (Figs 6 and 8), occurring at higher *P*/*P*_0_. The relationship between the transition point and muscle stiffness is highly temperature sensitive (Fig. S4B) and may be underpinned by temperature-dependent changes in internal viscosity and viscoelastic coupling within the myofilament lattice (Colombini et al., 2008; Minozzo and Rassier, 2010; Rassier and Månsson, 2024). This shift impacts the derived force–velocity relationship, resulting in a significantly higher plateau (Fig. 5). In addition, we also observed the appearance of muscle ‘give’ (Tomalka, 2023; Tomalka et al., 2017), previously termed the ‘yield point’ by Josephson and Stokes (1999) (Figs 6 and 8). Muscle ‘give’ has been observed in both mammalian (Kissane and Askew, 2024; Linari et al., 2004) and non-mammalian (Flitney and Hirst, 1978; Josephson and Stokes, 1999) muscles, across multiple structural scales, including skinned fibres (Linari et al., 2004; Weidner et al., 2022, 2024), *ex vivo* whole muscles (Askew and Kissane, 2026; Kissane and Askew, 2024) and *in situ* preparations (Joyce et al., 1969). This cross-scale consistency indicates that muscle ‘give’ is an intrinsic feature of active lengthening. While three-dimensional muscle architecture (and associated gearing) is known to influence concentric behaviour of muscle (Azizi et al., 2008; Eng et al., 2018), its role in eccentric contractions and in the manifestation of muscle ‘give’ are unknown. We have previously suggested that muscle ‘give’ arises through a temporal offset between the ATP-dependent and strain-dependent cross-bridge detachment process of phase-1, and the activated parallel elastic elements of phase-2 (Askew and Kissane, 2026). However, the mechanistic basis and functional significance of this phenomenon is unknown. Muscle ‘give’ is considered a velocity-dependent phenomenon (Josephson and Stokes, 1999; Tomalka, 2023; Weidner et al., 2022), which our data support. However, it also appears to be highly temperature sensitive (Figs 6 and 8). For example, Tomalka (2023) observed muscle ‘give’ at a relative lengthening velocity of 0.1 *V*/*V*_max_, in skinned fibres at 11°C. In contrast, at physiological temperatures (37°C), we did not observe muscle ‘give’ even at the highest relative stretch velocities (*V*/*V*_max_ ∼0.45). One possible explanation is that elevated temperatures enhance titin–thin filament interactions. Earlier engagement of these elements would increase the stiffness during lengthening and reduce the temporal offset between phase-1 and phase-2 processes, thereby suppressing the occurrence of muscle ‘give’. This does not rule out the occurrence of ‘give’ at core body temperature, as we may not have applied sufficiently high lengthening velocities to this muscle.

Finally, we demonstrate that the relationship between the rate of relative force development and stretch velocity during phase-2 is temperature sensitive. Here, the slope of this relationship is steeper at higher temperatures indicating greater stiffness of the muscle, as indicated by *Q*_10_ values exceeding 1: 2.25 for the 27°C to 37°C range. These observations suggest that elevated temperatures enhance titin–actin interactions, either by accelerating or by strengthening binding, thereby increasing stiffness through a higher proportion of titin molecules attached to the thin filament (Nishikawa et al., 2020).

### Generalisability across muscle types

Intrinsic differences in muscle architecture (e.g. relative fibre length and pennation angle) and myosin isoforms are likely to influence the sensitivity of the biphasic force response to starting length and temperature. Differences in myosin (Luff, 1981) and titin isoforms (Prado et al., 2005) across phenotypically distinct muscles might therefore be expected to produce different sensitivities to temperature. However, collated data from fast- and slow-twitch muscles from mouse (present study; Fig. S4; Askew and Kissane, 2026) suggest that such differences are limited for the eccentric force–velocity relationship across the muscles examined. Recent work has demonstrated that temperature sensitivity of the concentric force–velocity relationship is muscle specific and can vary regionally within an organism (Rummel et al., 2021, 2022); therefore, thermal dependence may not necessarily be uniform across all muscles, with phenotype potentially not the underpinning mechanism.

### Considerations of the characterisation of the eccentric force–velocity relationship

The eccentric force–velocity relationship is commonly modelled using a double hyperbolic relationship (Alcazar et al., 2019; Millard et al., 2013; Tomalka, 2023). The equation by Alcazar et al. (2019) currently provides the best fit to our data and includes two coefficients that define plateau height and curvature (Fig. S1). These coefficients provide a means of directly comparing eccentric muscle behaviour across different research domains (e.g. comparative muscle physiology and pathophysiological research). However, unlike concentric isotonic or isovelocity contractions in which force and velocity have a single corresponding value, during eccentric stretches force changes dynamically throughout the stretch. Consequently, the eccentric force–velocity relationship depends critically on the point during the ramp at which force is measured. Our approach has been to lengthen the muscle across the plateau of the force–length relationship (about *L*_0_), and record relative force over a length change spanning *L*_0_, corresponding to phase-2 (Kissane and Askew, 2024). While this approach provides experimental standardisation, the resulting eccentric force–velocity relationship fails to capture the dynamic nature of force development during active stretch and therefore does not accurately represent muscle behaviour, and may reduce the accuracy of force predictions in musculoskeletal models.

To address this limitation, we developed a multi-dimensional representation of eccentric contractions that maps muscle length, lengthening velocity and force, together with a heat map of the rate of relative force development (Figs 7 and 8). This 3D approach captures the interplay between phase-1 and phase-2 responses and incorporates the dynamic behaviour that cannot be represented in a single force–velocity relationship. By providing a framework that integrates both force and its rate of development across variation in velocity and length, this method offers a more physiologically realistic representation of muscle behaviour during active stretch. It also establishes a more rigorous foundation for interpreting experimental data and for incorporating eccentric muscle properties into musculoskeletal models (Yeo et al., 2023).

### Concluding remarks

In summary, by examining the effects of initial starting length and temperature on the eccentric force–velocity relationship, we demonstrate how cross-bridge detachment kinetics and parallel elastic components shape the dynamic force response of muscle during active stretch. Therefore, musculoskeletal models must incorporate the behaviours that arise from these underlying mechanisms, moving beyond traditional, simplified eccentric force–velocity relationships. Incorporating these dynamics will substantially improve the predictive accuracy of musculoskeletal models of movements involving energy dissipation by muscle fascicles, with important implications for understanding and predicting *in vivo* performance and injury risk.

## Supplementary Material

10.1242/jexbio.252267_sup1 Supplementary information
