# Supplementary material for: Starting length and temperature dependence of eccentric muscle force–velocity behaviour
Source: J Exp Biol. 2026 Jun 4;229(11):jeb252267. doi: 10.1242/jeb.252267 (PMC13286365; doi:10.1242/jeb.252267)
Supplement: Supplementary information [file jexbio-229-252267-s1.pdf]

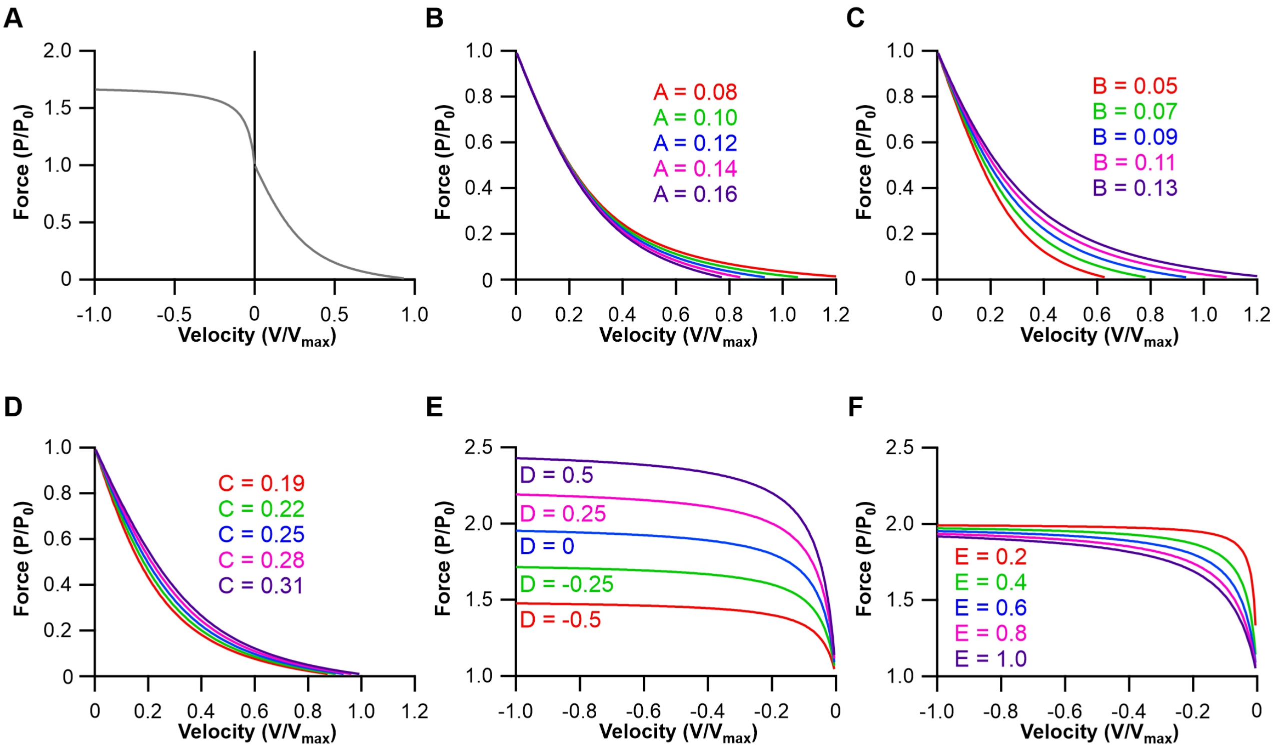

Fig. S1. Coefficients of the force-velocity fit equations. The concentric force- velocity relationship has been determined by fitting a hyperbolic-linear function from Marsh and Bennett (1986), while the eccentric force-velocity relationship has been fit with the hyperbolic equation described by Alcazar et al. (2019) (A). Changes in coefficient A (B) and B (C) appear to predominantly alter the curvature of the velocity estimate towards the estimates of V_{max}. While the C coefficient (D) appears to be closely associated with changes in curvature. While for the eccentric force-velocity relationship changes in coefficient D correspond with changes in the plateau height (E) while changes in the E coefficient vary the curvature of this relationship (F).

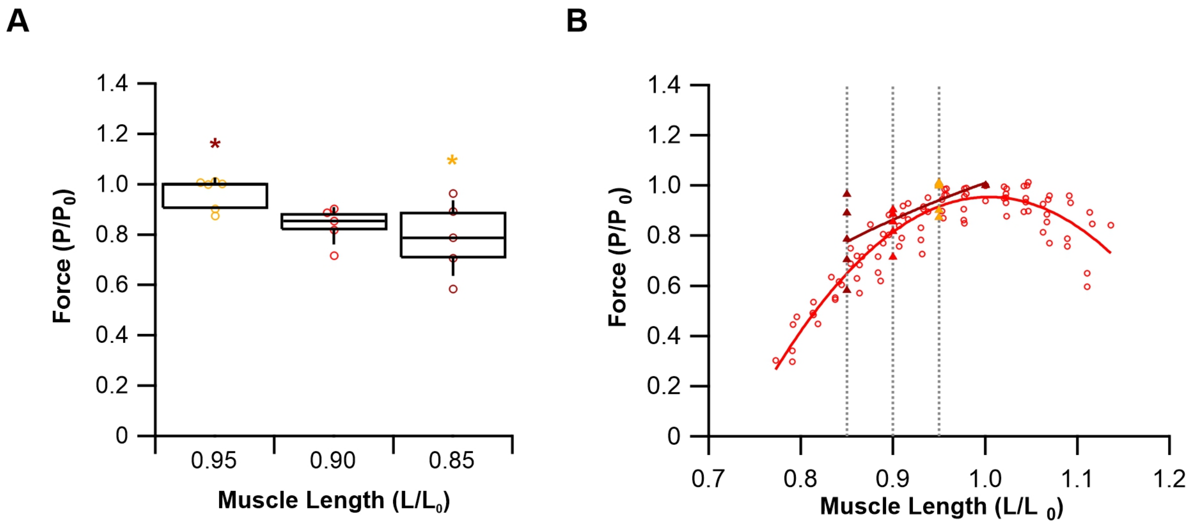

Fig. S2. The impact of starting length on tetanic force. The relative tetanic force produced at the beginning of the eccentric ramp (A) relative to tetanic force at L_0. Overlay of the soleus force-length relationship for the isometric twitch (unfilled circles) and the tetanus (filled triangles) (B). $0.95L/L_0$ (n=6), $0.90L/L_0$ (n=5), $0.85L/L_0$ (n=5).

Journal of Experimental Biology • Supplementary information

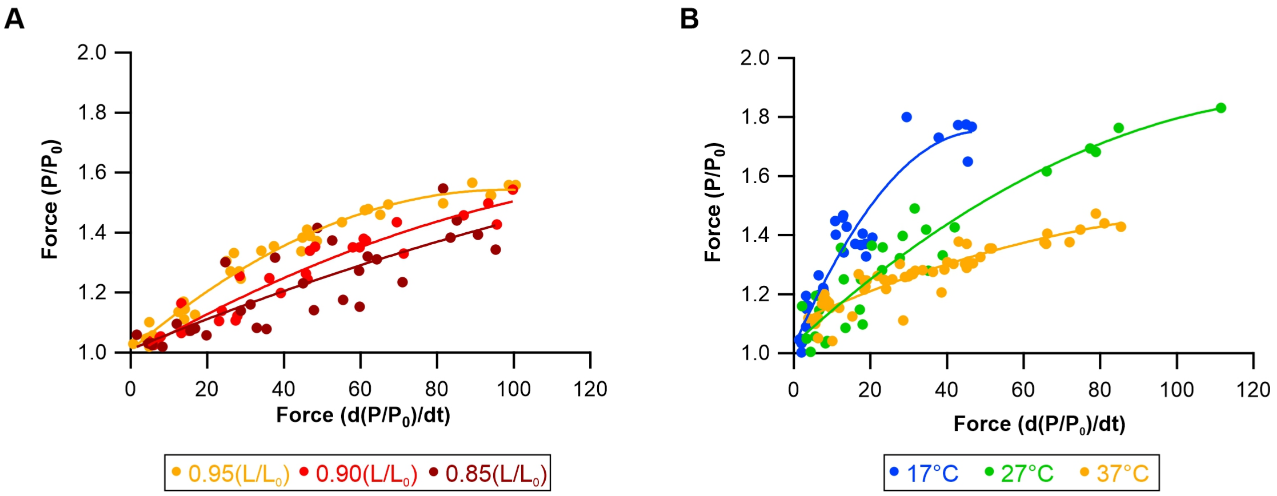

Fig. S3. The transition between Phase-1 and Phase-2. Here we present the absolute (P/P_0) transition point against the rate of force development during phase-1 (i.e. the stiffness) in response to altered starting length (A) and temperature (B). $0.95L/L_0$ (n=6), $0.90L/L_0$ (n=5), $0.85L/L_0$ (n=5), 17° C (n=6), 27°C (n=5), 37°C (n=8).

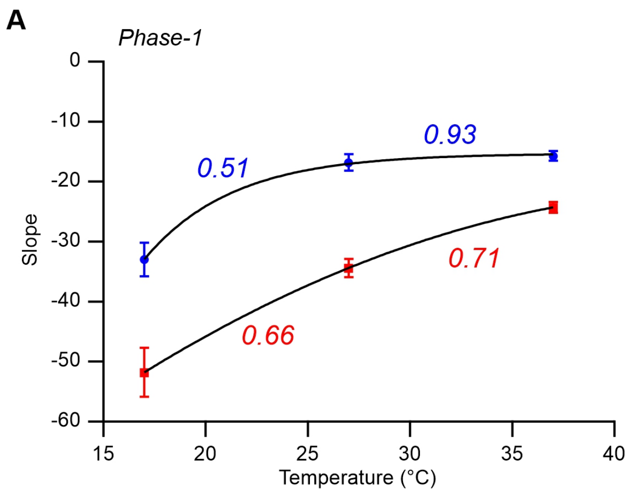

Fig. S4. There appears to be no clear phenotypic difference in the temperature sensitivity of the eccentric force-velocity relationship. The fast extensor digitorum longus (EDL, blue circles) data are taken from Askew & Kissane (2026) plot against the soleus (SOL, red squares) for phase-1 (A) and phase-2 (B) slopes. Note, the starting lengths for these two experiments differ, SOL (0.900-0.906 L/L_0) compared to the EDL (0.852-0.862 L/L_0) and likely underscores the difference in the phase-1 slopes seen at 37°C. Here the SOL Q_{10} for the 37-17°C range was 0.69 for phase-1 compared to 0.69 for the EDL, while the Q_{10} for the 37-17°C range across phase-2 was 1.42 for the SOL, compared to 1.38 for the EDL.

Journal of Experimental Biology • Supplementary information

Table S1. Linear regression model statistics and pairwise statistics for the slope between the rates of force development and velocity of muscle lengthening across phase-1 and phase-2 in response to altered starting length.

	**Slopes [95% confidence limits]**			**Model Fit**	
	**0.95 (L/L_0)**	**0.90 (L/L_0)**	**0.85 (L/L_0)**	**F**	**P value**
Phase-1 - Absolute Velocity vs. Temp	-33.2 [-35.5, -30.9] $^{\S,\#}$	-27.3 [-29.5, -25.1] *	-25.9 [-28.0, -23.8] *	405.4	<0.001
Phase-2 - Absolute Velocity vs. Temp	-3.70 [-4.36, -3.04]	-3.72 [-4.36, -3.08]	-4.47 [-5.08, -3.86]	97.79	<0.001

P value adjusted, Tukey method for comparing pairwise differences. * P<0.05 *vs. 0.95L/L₀* (n=6), § P<0.05 *vs. 0.90L/L₀* (n=5), # P<0.05 *vs. 0.85L/L₀* (n=5).

Table S2. Linear regression model statistics and pairwise statistics for the slope between the rates of force development and velocity of muscle lengthening across phase-1 and phase-2 in response to temperature.

	**Slopes [95% confidence limits]**			**Model Fit**	
	**17°C**	**27°C**	**37°C**	**F**	**P value**
Phase-1 - Absolute Velocity vs. Temp	-51.8 [-55.9, -47.8] $^{\S,\#}$	-34.4 [-35.9, -32.9] $^{*,\#}$	-24.3 [-25.2, -23.3] *,§	1209	<0.001
Phase-1 - Normalised Velocity vs. Temp	-95.9 [-103, -88.3] $^{\S,\#}$	-170.6 [-178, -163.4] *	-170.0 [-177, -163.2] *	1209	<0.001
Phase-2 - Absolute Velocity vs. Temp	-2.14 [-3.22, -1.05] $^{\#}$	-1.91 [-2.30, -1.52] $^{\#}$	-4.30 [-4.56, -4.04] *,§	337.4	<0.001
Phase-2 - Normalised Velocity vs. Temp	-3.95 [-5.96, -1.95] $^{\S,\#}$	-9.47 [-11.39, -7.56] $^{*,\#}$	-30.15 [-31.95, -28.35] *,§	337.4	<0.001

P value adjusted, Tukey method for comparing pairwise differences. * P<0.05 *vs.* 17°C (n=6), § P<0.05 *vs.* 27°C (n=5), # P<0.05 *vs.* 37°C (n=8).

Journal of Experimental Biology • Supplementary information
